# Gender-specific factors related to suicidal ideation among community-dwelling stroke survivors: The 2013 Korean Community Health Survey

**DOI:** 10.1371/journal.pone.0201717

**Published:** 2018-08-02

**Authors:** Mina Kim, Young-Hoon Lee

**Affiliations:** 1 Department of Nursing, Graduate School, Chonnam National University, Donggu, Gwangju, Republic of Korea; 2 Department of Counseling Psychology, Graduate School of Dongshin University, Naju, Jeonnam, Republic of Korea; 3 Department of Preventive Medicine & Institute of Wonkwang Medical Science, Wonkwang University School of Medicine, Iksan, Jeonbuk, Republic of Korea; 4 Regional Cardiocerebrovascular Center, Wonkwang University Hospital, Iksan, Jeonbuk, Republic of Korea; Stellenbosch University, SOUTH AFRICA

## Abstract

We assessed gender-specific factors associated with suicidal ideation among community-dwelling stroke survivors. In total, 4,322 stroke survivors who participated in the 2013 Korean Community Health Survey were included in the final analysis. Sociodemographic information, socio-family relationships, health behaviors, health status, and suicidal ideation were assessed using a standardized questionnaire. On fully adjusted analysis, suicidal ideation among males was more common in those who were widowed, rather than married (odds ratio [OR] 1.59, 95% confidence interval [CI] 1.03–2.47), those who rarely contacted neighbors (OR 1.50, 95% CI 1.10–2.06), current smokers (OR 1.54, 95% CI 1.03–2.29), and frequent drinkers (OR 1.54, 95% CI 1.05–2.24). Suicidal ideation among females was more common in older subjects, those with lower monthly household incomes, the unemployed (OR 1.75, 95% CI 1.21–2.53), and housewives/students (OR 1.46, 95% CI 1.06–2.03), those who rarely contacted friends (OR 1.43, 95% CI 1.12–1.82), and diabetics (OR 1.35, 95% CI 1.05–1.73). Perceived persistent high-level stress, depressive mood, poor self-rated health, and a diagnosis of depression were commonly associated with suicidal ideation in both genders. Gender differences should be considered by medical practitioners and community policymakers when seeking to prevent and manage suicidal ideation in stroke survivors.

## Introduction

Stroke is a major public health problem worldwide. In 2015, 8.97 million acute first strokes, 42.43 million cases of overall stroke, and 6.33 million stroke-related deaths occurred worldwide [1]. Although age-adjusted stroke mortality has tended to decrease gradually over recent years [2], the socioeconomic burden of stroke is continuously increasing because of an elevated incidence, attributable to the rapid increase in the number of elderly residents of Korea [3].

Previous studies have described an increased risk of suicide among stroke survivors [4,5]. Compared with subjects who had not suffered a stroke, those who had were found to be at increased risk of suicidal ideation and attempted suicide [6]. Thus, the identification of factors associated with suicidal ideation after stroke is an important clinical and public health issue. Recent studies have identified relevant risk factors for suicidal ideation in stroke patients [7–11]. However, these studies focused principally on clinical characteristics, including only some sociodemographic factors as independent variables, and many studies evaluated small samples of stroke patients.

Our previous study of a community-dwelling general population with suicidal ideation revealed gender differences in the risk factors associated with suicide attempts [12]. Similarly, another study from our research group identified gender-specific factors associated with the use of mental health services for suicidal ideation in a community-dwelling general population [13]. Due to the significant roles that adults play in their family and society and the existence of gender role differences, the impact of suicidal ideation should be independently analyzed based on gender. However, little information is available on gender differences in factors related to suicidal ideation among stroke survivors. Previous studies have shown that suicide is more frequent among female stroke victims than among male stroke victims [10]. The analysis of relevant risk factors by gender may help to identify subgroups of stroke patients at increased risk of suicide and allow the development of gender-specific preventative interventions for high-risk stroke survivors. An effective strategy for suicide prevention can begin after identifying gender-specific factors related to suicidal ideation in the early stages of this process. Therefore, we sought to identify gender-specific factors, including sociodemographic factors, socio-family relationships, health behaviors, and health status parameters, associated with suicidal ideation among community-dwelling stroke survivors in a large representative sample of the Korean population. Unlike our previous studies of individuals with suicidal ideation from the general population [12,13], the present study aimed to identify gender differences in factors that increase suicidal ideation among stroke survivors.

## Methods

### Design and samples

Cross-sectional data from the 2013 Korean Community Health Survey (KCHS) conducted by the Korea Centers for Disease Control and Prevention (KCDC) were used in this study. The KCHS is a nationwide survey conducted every year since 2008 by trained surveyors using computer-assisted personal interviewing methods. Multistage, stratified random sampling was used to select representative households in 253 local communities, based on information obtained from resident registrations. An average of 900 subjects in each local community were surveyed. A total of 228,781 individuals aged 19 years or older participated in the 2013 survey. Of these, 4,560 (2.0%) had been diagnosed with stroke before the survey. After excluding participants for whom any sociodemographic, socio-family relationship, health behavior, and/or health status data were missing, 4,322 stroke survivors (2,205 males and 2,117 females) were included in the final analysis.

This study was conducted in accordance with all relevant guidelines of the Declaration of Helsinki. Our study protocol was approved by the Institutional Review Board of the Wonkwang University Hospital (IRB number: WKUH 2017-09-001).

### Measures

The dependent variable was gender-specific suicidal ideation among stroke survivors. Suicidal ideation has been defined as having thoughts of wanting to die in the past year. Data on four sets of potential risk factors for suicidal ideation were collected using a standardized questionnaire: sociodemographic factors, socio-family relationships, health behaviors, and health status. A detailed description of the variables is provided in Table 1.

**Table 1:** Summary of variables.

Variables	Question	Category
**Outcome variable**		
Suicidal ideation	Have you ever thought of wanting to die in the past year?	Never or ever
**Sociodemographic factors**		
Age group	What is your age?	19–49, 50–64, 65–79, or ≥80 years
Residential region	Is your place of residence urban or rural?	Urban or rural
Marital status	Have you ever been married (including a common law marriage)? What is your current marital status?	Never married, married, divorced/separated, or widowed
Educational level	Where did you go to school? Did you graduate from school?	Non-formal education, primary school, middle or high school, or college or higher
Monthly household income	What was your average monthly household income in the past year, including wages, real estate income, pensions, interest, government subsidies, and allowances for relatives or children?	≤1, 1.01–2, 2.01–4, or ≥4.01 million KRW
Employment status	What occupation are you currently engaged in?	Employed, unemployed, or housewife/student
National Basic Livelihood Security status	Does your household currently receive National Basic Livelihood Security?	Recipient or non-recipient
**Socio-family relationships**		
Family contact	How often do you see or contact your closest relative (family member)?	<1 or ≥1 time per month
Neighbor contact	How often do you see or contact your closest neighbors?	<1 or ≥1 time per month
Friend contact	How often do you see or contact your most frequently contacted friends?	<1 or ≥1 time per month
Religious activity	Do you regularly engage in religious activities at least once a month?	<1 or ≥1 time per month
Friendship activity	Do you regularly engage in friendship activities at least once a month?	<1 or ≥1 time per month
Leisure activity	Do you regularly engage in leisure activities at least once a month?	<1 or ≥1 time per month
Charitable activity	Do you regularly engage in charitable activities at least once a month?	<1 or ≥1 time per month
**Health behaviors and health status**		
Smoking status	Have you smoked more than 100 cigarettes during your life? Do you smoke now?	Never smoker, former smoker, or current smoker
Frequency of alcohol use	Have you been drinking for the last year? How often do you drink alcohol?	None, ≤1, 2–3, or ≥4 times per week
Moderate physical activity		≤2 or ≥3 times per week
Walking activity	How many days did you walk for at least 10 minutes at a time in the last week?	≤2 or ≥3 times per week
Sleep duration	How many hours a day do you usually sleep?	≤6, 7–8, or ≥9 h per day
Perceived usual stress level	How often do you feel stressed in your usual life?	High (very often or often) or low (rarely or almost never)
Experience of depressive mood	Have you ever felt sad or desperate for more than 2 weeks in a row during the past year?	No or yes
Self-rated health	What do you think about your health?	Good, fair, or poor
Diagnosis of hypertension	Have you been diagnosed with hypertension?	Never or ever
Diagnosis of diabetes	Have you been diagnosed with diabetes?	Never or ever
Diagnosis of depression	Have you been diagnosed with depression?	Never or ever

The sociodemographic factors included age group, residential region, marital status, educational level, monthly household income, employment status, and National Basic Livelihood Security System status. The socio-family relationships evaluated included family contact, neighbor contact, friend contact, religious activity, friendship activity, leisure activity, and charity activity, classified as less or more than once a month. Health behaviors included smoking status, frequency of alcohol use, moderate physical activity, walking activity, and sleep duration. Health status variables included the perceived usual level of stress, experience of a depressive mood, and self-rated health. Hypertension, diabetes, and depression were classified as having been diagnosed or never diagnosed.

### Statistical analysis

All analyses were performed separately for males and females. The characteristics of the stroke survivors were compared based on the presence or absence of suicidal ideation using the chi-squared test. After adjusting for all evaluated covariates (sociodemographic factors, socio-family relationships, health behaviors, and health status), the odds ratio (OR) and 95% confidence interval (CI) for suicidal ideation associated with each factor were estimated via multivariate logistic regression analysis. All statistical analyses were performed with the aid of SPSS Statistics version 22.0 for Windows (IBM Co.; Armonk, NY, USA). A *p* value less than 0.05 was considered to indicate statistical significance.

## Results

Of the 2,205 males and 2,117 females who had been diagnosed with stroke, 429 (19.5%) males and 610 (28.8%) females had experienced suicidal ideation within the past year (Table 2).

**Table 2:** Suicidal ideation in and sociodemographic characteristics of stroke survivors by gender.

Variable	Males (*n* = 2,205)		Females (*n* = 2,117)		*P*
Suicidal ideation					<0.001
Never	1,776	(80.5)	1,507	(71.2)	
Ever	429	(19.5)	610	(28.8)	
Age group					<0.001
19–49 years	99	(4.5)	50	(2.4)	
50–64 years	540	(24.5)	405	(19.1)	
65–79 years	1,273	(57.7)	1,310	(61.9)	
≥80 years	293	(13.3)	352	(16.6)	
Residential region					0.009
Urban	930	(42.2)	810	(38.3)	
Rural	1,275	(57.8)	1,307	(61.7)	
Marital status					<0.001
Married	1,858	(84.3)	1,016	(48.0)	
Never married	55	(2.5)	15	(0.7)	
Divorced or separated	143	(6.5)	92	(4.3)	
Widowed	149	(6.8)	994	(47.0)	
Educational level					<0.001
Non-formal education	184	(8.3)	783	(37.0)	
Primary school	743	(33.7)	913	(43.1)	
Middle or high school	1,008	(45.7)	375	(17.7)	
College and higher	270	(12.2)	46	(2.2)	
Monthly household income					<0.001
≤1 million KRW	1,191	(54.0)	1,263	(59.7)	
1.01–2 million KRW	483	(21.9)	351	(16.6)	
2.01–4 million KRW	340	(15.4)	325	(15.4)	
≥4.01 million KRW	191	(8.7)	178	(8.4)	
Employment status					<0.001
Employed	790	(35.8)	480	(22.7)	
Unemployed	1,407	(63.8)	605	(28.6)	
Housewife or student	8	(0.4)	1,032	(48.7)	
National Basic Livelihood Security status					0.700
Non-recipient	1,997	(90.6)	1,910	(90.2)	
Recipient	208	(9.4)	207	(9.8)	

Data are presented as numbers (percentages).

Tables 2, 3 and 4 show the distributions of sociodemographic factors, socio-family relationships, health behaviors, and health status by gender in stroke survivors. Those aged 65–79 years (males: 57.7%, females: 61.9%) and those with a monthly household income of ≤ 1 million KRW (males: 54.0%, females: 59.7%) accounted for a majority of both men and women. There were more rural residents (males: 57.8%, females: 61.7%) than urban residents, and the majority of males were married (84.3%), whereas females were more likely to be married (48.0%) or widowed (47.0%). More than half the males went beyond middle school in terms of educational achievement, whereas approximately 80% of females did not graduate from primary school. In terms of employment status, more males were unemployed (63.8%), and more females were housewives/students (48.7%; Table 2).

**Table 3:** Socio-family relationships in stroke survivors by gender.

Variable	Males		Females		*P*
Family contact					0.090
<1 time per month	436	(19.8)	376	(17.8)	
≥1 time per month	1,769	(80.2)	1,741	(82.2)	
Neighbor contact					<0.001
<1 time per month	496	(22.5)	332	(15.7)	
≥1 time per month	1,709	(77.5)	1,785	(84.3)	
Friend contact					<0.001
<1 time per month	855	(38.8)	996	(47.0)	
≥1 time per month	1,350	(61.2)	1,121	(53.0)	
Religious activity					<0.001
<1 time per month	1,715	(77.8)	1,348	(63.7)	
≥1 time per month	490	(22.2)	769	(36.3)	
Friendship activity					<0.001
<1 time per month	1,236	(56.1)	1,395	(65.9)	
≥1 time per month	969	(43.9)	722	(34.1)	
Leisure activity					<0.001
<1 time per month	1,941	(88.0)	1,968	(93.0)	
≥1 time per month	264	(12.0)	149	(7.0)	
Charitable activity					0.430
<1 time per month	2,119	(96.1)	2,044	(96.6)	
≥1 time per month	86	(3.9)	73	(3.4)	

Data are presented as numbers (percentages).

**Table 4:** Health behaviors and health status of stroke survivors by gender.

Variable	Males		Females		*P*
Smoking status					<0.001
Never-smoker	401	(18.2)	1,976	(93.3)	
Former smoker	1,307	(59.3)	82	(3.9)	
Current smoker	497	(22.5)	59	(2.8)	
Frequency of alcohol use					<0.001
None	1,180	(53.5)	1,607	(75.9)	
≤1 time per week	533	(24.2)	421	(19.9)	
2–3 times per week	207	(9.4)	57	(2.7)	
≥4 times per week	285	(12.9)	32	(1.5)	
Moderate physical activity					0.012
≤2 times per week	1,827	(82.9)	1,813	(85.6)	
≥3 times per week	378	(17.1)	304	(14.4)	
Walking activity					0.003
≤2 times per week	969	(43.9)	1,025	(48.4)	
≥3 times per week	1,236	(56.1)	1,092	(51.6)	
Sleep duration					<0.001
≤6 h per day	893	(40.5)	1,057	(49.9)	
7–8 h per day	1,070	(48.5)	882	(41.7)	
≥9 h per day	242	(11.0)	178	(8.4)	
Perceived usual stress level					<0.001
Low	1,615	(73.2)	1,391	(65.7)	
High	590	(26.8)	726	(34.3)	
Experience of depressive mood					<0.001
No	1,998	(90.6)	1,789	(84.5)	
Yes	207	(9.4)	328	(15.5)	
Self-rated health					<0.001
Good	215	(9.8)	144	(6.8)	
Fair	505	(22.9)	365	(17.2)	
Poor	1,485	(67.3)	1,608	(76.0)	
Diagnosis of hypertension					0.001
Never	742	(33.7)	616	(29.1)	
Ever	1,463	(66.3)	1,501	(70.9)	
Diagnosis of diabetes					0.235
Never	1,631	(74.0)	1,532	(72.4)	
Ever	574	(26.0)	585	(27.6)	
Diagnosis of depression					<0.001
Never	2,091	(94.8)	1,896	(89.6)	
Ever	114	(5.2)	221	(10.4)	

Data are presented as numbers (percentages).

In terms of interpersonal contact, the family contact rate (80.2%) was highest and the friend contact rate (61.2%) was lowest in males, whereas the neighbor contact rate (84.3%) was highest and the friend contact rate (53.0%) was lowest in females. In terms of social activities, the rate of participation in friendship activities (43.9%) was highest and the rate of participation in charitable activity (3.9%) was lowest in males. The participation rate in religious activity (36.3%) was highest and the participation rate in charitable activity (3.4%) was lowest in females (Table 3).

Regarding health behaviors, there were more male former smokers (59.3%), whereas almost all females were never-smokers (93.3%). Non-drinkers constituted majorities of both males (53.5%) and females (75.9%), and the rates of moderate physical activity (≥ 3 times/week) and walking activity (≥ 3 times/week) were 17.1% and 56.1% in males and 14.4% and 51.6% in females, respectively. As for sleep duration, males were most likely to sleep 7–8 h/day (48.5%) and females were most likely to sleep ≤ 6 h/day (49.9%). Of the health status parameters, the proportions of subjects who perceived that they usually experienced a high level of stress and a depressed mood were 26.8% and 9.4% in males and 34.3% and 15.5% in females, respectively. Majorities of both genders perceived their health to be poor (males: 67.3%, females: 76.0%). The rates of people diagnosed with hypertension, diabetes, and depression were 66.3%, 26.0%, and 5.2% in males and 70.9%, 27.6%, and 10.4% in females, respectively (Table 4).

Table 5 shows the fully adjusted statistics for gender-specific relationships between suicidal ideation and related factors. We found a significant trend toward increased suicidal ideation with age in females, but not in males. Compared with those aged 19–49 years, the ORs for suicidal ideation among those aged 50–64, 65–79, and ≥80 years increased by 2.42- (95% CI 0.94–6.25), 2.71- (95% CI 1.03–7.12), and 3.30-fold (95% CI 1.20–9.07) among females. Compared with those who were married, suicidal ideation was significantly more common among those who were widowed, but only among males (OR 1.59, 95% CI 1.03–2.47). Suicidal ideation was significantly more frequent among females with monthly household incomes of 1.01–2 million won (OR 1.97, 95% CI 1.13–3.41) and ≤1 million won (OR 2.06, 95% CI 1.25–3.41) compared with those with incomes ≥ 4.01 million won. Unlike the situation in females, we found no significant association between monthly household income and suicidal ideation among males. Females who were unemployed (OR 1.75, 95% CI 1.21–2.53) or housewives/students (OR 1.46, 95% CI 1.06–2.03) exhibited higher ORs for suicidal ideation, whereas we found no significant association between employment status and suicidal ideation in males. No significant association was observed between residential region, educational level, or National Basic Livelihood Security System recipient status and suicidal ideation in males or females. A lack of neighbor contact was associated positively with suicidal ideation among males (OR 1.50, 95% CI 1.10–2.06), whereas a lack of friend contact was associated with suicidal ideation among females (OR 1.43, 95% CI 1.12–1.82).

**Table 5:** Fully-Adjusted odds ratios and 95% confidence intervals for the associations between study covariates and suicidal ideation in stroke survivors: Multivariate logistic regression analysis.

Variable	Males		Females	
**Sociodemographic factors**				
Age group (reference: 19–49 years)				
50–64 years	0.78	(0.37–1.63)	2.42	(0.94–6.25)
65–79 years	1.28	(0.60–2.71)	2.71*	(1.03–7.12)
≥80 years	1.05	(0.46–2.40)	3.30*	(1.20–9.07)
Rural (reference: urban)	1.09	(0.82–1.45)	1.28	(0.99–1.67)
Marital status (reference: married)				
Never married	0.59	(0.22–1.57)	2.51	(0.68–9.33)
Divorced or separated	1.45	(0.89–2.38)	1.54	(0.88–2.67)
Widowed	1.59*	(1.03–2.47)	1.23	(0.95–1.59)
Educational level (reference: college or higher)				
Middle or high school	0.77	(0.50–1.17)	0.66	(0.26–1.67)
Primary school	1.00	(0.63–1.57)	0.66	(0.26–1.68)
Non-formal education	1.19	(0.68–2.09)	0.68	(0.26–1.77)
Monthly household income (reference: ≥4.01 million KRW)				
2.01–4 million KRW	0.68	(0.37–1.24)	1.49	(0.85–2.60)
1.01–2 million KRW	1.16	(0.67–2.01)	1.97*	(1.13–3.41)
≤1 million KRW	1.28	(0.76–2.16)	2.06*	(1.25–3.41)
Employment status (reference: employed)				
Unemployed	1.29	(0.94–1.78)	1.75*	(1.21–2.53)
Housewife or student	0.56	(0.09–3.33)	1.46*	(1.06–2.03)
Recipient of National Basic Livelihood Security (reference: non-recipient)	1.00	(0.65–1.52)	1.18	(0.81–1.73)
**Socio-family relationships**				
Family contact < 1 time per month (reference: ≥1 time)	1.12	(0.83–1.51)	1.07	(0.80–1.44)
Neighbor contact < 1 time per month (reference: ≥1 time)	1.50*	(1.10–2.06)	0.81	(0.58–1.13)
Friend contact < 1 time per month (reference: ≥1 time)	1.22	(0.92–1.61)	1.43*	(1.12–1.82)
Religious activity < 1 time per month (reference: ≥1 time)	1.04	(0.77–1.42)	1.18	(0.92–1.51)
Friendship activity < 1 time per month (reference: ≥1 time)	1.13	(0.84–1.52)	1.24	(0.95–1.63)
Leisure activity < 1 time per month (reference: ≥1 time)	0.75	(0.47–1.20)	1.07	(0.63–1.80)
Charitable activity < 1 time per month (reference: ≥1 time)	1.04	(0.47–2.30)	0.65	(0.32–1.33)
**Health behaviors and health status**				
Smoking status (reference: never smoker)				
Former smoker	1.08	(0.77–1.52)	1.52	(0.87–2.64)
Current smoker	1.54*	(1.03–2.29)	1.23	(0.66–2.32)
Frequency of alcohol use (reference: non-drinkers)				
≤1 time per week	1.10	(0.79–1.52)	1.21	(0.91–1.63)
2–3 times per week	1.44	(0.91–2.28)	1.03	(0.51–2.09)
≥4 times per week	1.54*	(1.05–2.24)	0.63	(0.22–1.80)
Moderate physical activity ≤ 2 times per week (reference: ≥3 times)	1.09	(0.76–1.57)	1.12	(0.79–1.58)
Walking activity ≤ 2 times per week (reference: ≥3 times)	0.93	(0.72–1.21)	0.94	(0.74–1.19)
Sleep duration (reference: 7–8 h per day)				
≤6 h per day	0.92	(0.70–1.20)	1.15	(0.91–1.46)
≥9 h per day	1.11	(0.76–1.63)	0.93	(0.61–1.43)
Perceived high usual stress level (reference: low level)	2.85*	(2.20–3.69)	3.01*	(2.37–3.81)
Experienced depressive mood (reference: not experienced)	6.00*	(4.21–8.55)	7.05*	(5.20–9.55)
Self-rated health (reference: good)				
Fair	1.66	(0.87–3.16)	1.37	(0.71–2.66)
Poor	2.99*	(1.65–5.44)	1.99*	(1.09–3.63)
Ever diagnosed with hypertension (reference: never diagnosed)	1.09	(0.83–1.42)	0.98	(0.76–1.27)
Ever diagnosed with diabetes (reference: never diagnosed)	0.99	(0.75–1.31)	1.35*	(1.05–1.73)
Ever diagnosed with depression (reference: never diagnosed)	2.59*	(1.61–4.15)	2.09*	(1.47–2.99)

*P<0.05.

Compared with never-smokers, suicidal ideation was significantly higher among current smokers, but only in males (OR 1.54, 95% CI 1.03–2.29). Compared with males who never drank alcohol, males who drank alcohol ≥4 times per week had a significantly higher rate of suicidal ideation (OR 1.54, 95% CI 1.05–2.24). Psychological indices, such as perceived usual stress level, experience of depressive mood, and a diagnosis of depression, were associated significantly with suicidal ideation among participants of both genders. Compared with participants with low stress levels, suicidal ideation was significantly higher among those with high stress levels in males (OR 2.85, 95% CI 2.20–3.69) and females (OR 3.01, 95% CI 2.37–3.81). Compared with participants without experience of a depressive mood, suicidal ideation was significantly higher among those with such an experience in males (OR 6.00, 95% CI 4.21–8.55) and females (OR 7.05, 95% CI 5.20–9.55). A history of depression diagnosis was associated significantly with suicidal ideation in males (OR 2.59, 95% CI 1.61–4.15) and females (OR 2.09, 95% CI 1.47–2.99). Suicidal ideation was significantly more common among those with poor self-rated health compared with good self-rated health for both genders (OR 2.99, 95% CI 1.65–5.44 in males and OR 1.99, 95% CI 1.09–3.63 in females). Female stroke survivors who had ever been diagnosed with diabetes exhibited higher-level suicidal ideation (OR 1.35, 95% CI 1.05–1.73).

## Discussion

We defined gender differences in factors increasing suicidal ideation among community-dwelling stroke survivors. Among males, suicidal ideation was more common in those who were widowed, rather than married, those who rarely contacted neighbors, current smokers, and frequent drinkers. On the other hand, among females, older subjects, those with lower monthly household incomes, those who were unemployed or who were housewives/students, those who rarely contacted friends, and diabetics exhibited higher-level suicidal ideation. The present findings also revealed that perceived usual stress, depressive mood, self-rated health, and depression were associated with suicidal ideation in both genders.

We found that age was associated positively with the risk of suicidal ideation in females, but not in males. The relevance of age in terms of suicidal ideation in stroke survivors is not clear because the link has been evaluated differently in various studies. Suicidal ideation was more common in younger stroke patients than in those aged 65 years and older [11]. In contrast, older age was a significant risk factor for suicidal ideation in stroke patients [7]. However, some studies showed that age was not associated with the extent of suicidal ideation after stroke [8,9]. As the associations among gender, age, and suicidal ideation after stroke thus remain undetermined, further research is required. Previous studies have observed a significant association between marital status and the risk of suicide. A recent meta-analysis showed that suicidal ideation was less likely in stroke survivors who were married (OR 0.63) [14]. We also found that widowed males were more likely than married males to exhibit suicidal ideation (OR 1.59), whereas no relationship between marital status and suicidal ideation was evident in females. Why only widowed males who have had strokes exhibit higher-level suicidal ideation is difficult to explain. One possible explanation is that the impact of bereavement differs between genders, although it may be common in males and females. Regardless of marital status, females reported better support networks characterized by more meaningful friendships, whereas males reported networks with less meaningful friendships and less beneficial and supportive social bonds [15]. Therefore, females are more likely to be supported by meaningful social and familial networks, whereas males lack safety-imparting social connections after bereavement [16].

In general, employment and income levels may have a greater effect on suicidal behavior among males than among females, particularly in a more traditional society where males are the “breadwinners” and/or male identity is intertwined with occupation. One meta-analysis showed that stroke survivors who were employed were at lesser risk of suicidal ideation (OR 0.57) [14]. We also found that unemployed female stroke survivors were more likely than their employed counterparts to exhibit suicidal ideation (OR 1.75). In addition, we found that monthly household income was associated significantly with suicidal ideation among female stroke survivors, being about twice as high in the lowest income group compared with the highest income group. However, no relationship between employment status or household income and suicidal ideation was evident in males. In general, adults in poor financial circumstances are more likely than those in good financial circumstances to exhibit suicidal ideation [17]. Our previous study conducted in a community-dwelling general population showed that household income was associated negatively with suicide attempts in males, but not in females [12]; this finding differs from the results of the present study. Here, we found that socioeconomic parameters, such as employment and income level, were associated significantly with suicidal ideation after stroke only in females. More economic support for female stroke patients is needed to prevent suicide. Community-based stroke survivors are predominantly elderly; whereas elderly males often have spouses, elderly females are typically widowed and live alone. Thus, unlike young adults in the general population, the impact of unemployment or low household income may be greater for females than for males among elderly stroke survivors.

Strong connections with family and the community may protect against suicide, especially in those who have suffered severe diseases, such as stroke. Family conflict is associated with more suicidal ideation and suicide attempts in the general population [18]. Meanwhile, post-stroke care programs improve the post-stroke management skills of family caregivers as well as enhance the functional status and reduce the complications of post-stroke patients [19]. Additionally, social support is associated with lower-level suicidal ideation and fewer suicide attempts [20]. In the present study, family contact was not associated with suicidal ideation among stroke survivors of either gender. Rather, neighbor contact in males and friend contact in females were associated positively with suicidal ideation of stroke survivors. Loneliness, an emotional condition that follows social isolation, is associated with an increased risk of stroke [21]. Loneliness also increases the risk of suicidal ideation in those in middle to late adulthood. Stroke survivors who are lonely and socially isolated may be at greater risk of suicidal ideation, and thus require mental and social support.

Suicidal behavior tends to occur in conjunction with other health behaviors rather than alone [22]. In the general population, suicide levels are higher among smokers and risky alcohol consumers [23]. Smoking and alcohol consumption have also been associated with suicide attempts [24]. We found that current smoking and frequent alcohol consumption were associated significantly with suicidal ideation only in male stroke patients. In Korea, males generally smoke and drink much more than do females, but these behaviors decrease with age in both genders [12]. Stroke usually affects older subjects, so female stroke survivors have much lower smoking and drinking rates than the general population. Furthermore, as females exhibit very low rates of smoking and drinking after stroke, unhealthy behaviors may not be related to suicidal ideation in females. However, it remains uncertain whether smoking status/drinking frequency, which were significantly associated with suicidal ideation in the present study, are the cause of suicidal ideation. Unlike sociodemographic factors, health behaviors can be interpreted as having a reciprocal relationship with suicidal ideation. Although the present results suggest that current smoking and frequent drinking may increase the risk of suicidal ideation, the causal relationship may also operate in the opposite direction (reciprocal links between smoking/drinking and suicidal ideation).

Regular screening for depression and suicidal ideation in stroke survivors is important. Meta-analyses have shown that the prevalence of post-stroke depression was 29% and the cumulative incidence thereof was 39–52% within 5 years after stroke onset [25]. The prevalence of depression was lower in the present study than in previous studies [25] because of differences in the study populations, stroke stage, and disease severity; females experienced twice as many strokes as males (5.2% for males and 10.4% for females). However, in the present study, experience of a depressive mood and a diagnosis of depression were associated strongly with suicidal ideation in stroke patients of both genders, with no difference apparent between genders. As we targeted community-dwelling stroke patients, we likely included more cases of chronic-phase than acute-phase stroke, and, therefore, more subjects who had experienced milder strokes than those who had been hospitalized with acute-phase strokes. Depression developing after stroke was associated significantly with functional disability, cognitive impairment, and increased mortality [26]. Recent studies have shown that active pharmacological treatment of depression can improve motor and cognitive recovery and increase long-term survival in depressed patients who have suffered strokes [27,28]. Therefore, regular clinical monitoring by hospitals and ongoing community public health programs are recommended to detect and treat depression in stroke patients. The prevention and early treatment of post-stroke depression should be a major component of a comprehensive program to reduce the risk of suicide-related behaviors [14].

Our study has certain limitations. First, causal relationships cannot be assessed using a cross-sectional design. Whether some factors considered to be relevant were actually in play prior to the development of suicidal ideation is thus uncertain. Second, information on such ideation was collected only from those living in the community after stroke. Thus, we could not evaluate the suicidal ideation of stroke patients admitted to hospital for acute or long-term care because they had experienced severe strokes. Therefore, the impact of stroke on suicidal ideation may have been underestimated. Third, information biases (such as recall bias) may be in play because information on suicidal ideation was collected retrospectively via self-reporting. Fourth, clinical data, such as stroke severity, functional impairment, complications, and time since the stroke, were not evaluated. Despite these limitations, the present study has several strengths. We evaluated data from a nationally representative sample of the general population, including a large number of community-dwelling stroke survivors. In addition, multiple covariates, such as sociodemographic parameters, socio-family relationships, health behaviors, and health status, were investigated simultaneously, by gender.

## Conclusions

The present study found that factors related to suicidal ideation differed by gender in stroke survivors. As a result, gender differences should be considered when medical practitioners and policymakers seek to prevent and manage suicidal ideation among community-dwelling stroke patients. It will be important to develop strategies for coping with common risk factors for suicidal ideation among both male and female stroke survivors, but different approaches to suicide prevention should be established based on the heterogeneous risk factors related to gender. Male stroke survivors, particularly those who are widowed and have little contact with their neighbors, need active social support. Additionally, health monitoring and interventions designed to change the behaviors of people who smoke and frequently drink will be needed. On the other hand, in female stroke survivors, especially those who are elderly, unemployed, and low-income, require economic support to prevent suicide. Additionally, more social outreach efforts are needed by those who have little contact with friends. Further longitudinal studies are needed to define the gender-specific causal relationships between suicidal ideation and associated factors.
